# Supplementary material for: Organization of the gravity-sensing system in zebrafish
Source: Nat Commun. 2022 Aug 27;13:5060. doi: 10.1038/s41467-022-32824-w (PMC9420129; doi:10.1038/s41467-022-32824-w)
Supplement: Supplementary file 3 — Description of Additional Supplementary Files [file 41467_2022_32824_MOESM3_ESM.pdf]

File name: Supplementary Data 1

Description: A table of connectivity from utricular hair cells to utricular afferents. Numbers in each cell represent the number of ribbon synapses from a hair cell onto an afferent. Table also includes the computed tuning of each hair cell in radians.

File name: Supplementary Data 2

Description: A table of connectivity from utricular afferents to identified central neurons in the brainstem. Numbers indicate the number of synaptic contacts. Where relevant, distinct release sites are counted as distinct contacts, but these are interpreted and less straightforward to quantify than ribbon synapses. Note that there are many afferent contacts onto central neurons not included in this table because they have not been reconstructed or identified. For example, some commissural neurons of unclear identity are not included. Table also includes the computed tuning of each afferent in radians.

File name: Supplementary Movie 1

Description: The 3D reconstructed utricular circuits in the larval zebrafish, related to Figure 1. Rendering of the utricular hair cells (gray), afferents (greens), Mauthner cell (black), and central vestibular neurons (oranges and blues) to show the entire gravity-sensing system. Utricles and eyes (gray volumes) are included for reference.

File name: Supplementary Movie 2

Description: The 3D reconstruction of utricular afferents and the utricular macula, related to Figure 2. Utricular afferent neurons are colorized according to rostrocaudal position as in Fig. 2d. The positioning of afferent somata reflects a systematic innervation pattern from the afferents to the hair cells.

File name: Supplementary Movie 3

Description: The 3D reconstruction of Mauthner and utricular commissural circuits, related to Figure 3. The right Mauthner (blue) and vestibular commissural (red) neurons receive projections from afferents that innervate the medial side of LPR (blue and yellow, tuned to ipsilateral tilt) and the lateral side of LPR (red, tuned to contralateral tilt). Note that the contralateral tilt (red) afferent axons exhibit a different trajectory in the brainstem than the ipsilateral tilt afferents.

File name: Supplementary Movie 4

Description: The 3D reconstruction of the superior vestibular and tangential circuits, related to Figure 4.
