# Supplementary material for: Organization of the gravity-sensing system in zebrafish
Source: Nat Commun. 2022 Aug 27;13:5060. doi: 10.1038/s41467-022-32824-w (PMC9420129; doi:10.1038/s41467-022-32824-w)
Supplement: Supplementary file 10 — Reporting Summary [file 41467_2022_32824_MOESM10_ESM.pdf]

nature portfolio

Corresponding author(s): Bagnall, M.W.

Last updated by author(s): Jun 30, 2022

Reporting Summary

Nature Portfolio wishes to improve the reproducibility of the work that we publish. This form provides structure for consistency and transparency in reporting. For further information on Nature Portfolio policies, see our Editorial Policies and the Editorial Policy Checklist.

Statistics

For all statistical analyses, confirm that the following items are present in the figure legend, table legend, main text, or Methods section.

n/a	Confirmed	
☐	☒	The exact sample size (*n*) for each experimental group/condition, given as a discrete number and unit of measurement
☐	☒	A statement on whether measurements were taken from distinct samples or whether the same sample was measured repeatedly
☐	☒	The statistical test(s) used AND whether they are one- or two-sided<br>*Only common tests should be described solely by name; describe more complex techniques in the Methods section.*
☒	☐	A description of all covariates tested
☐	☒	A description of any assumptions or corrections, such as tests of normality and adjustment for multiple comparisons
☐	☒	A full description of the statistical parameters including central tendency (e.g. means) or other basic estimates (e.g. regression coefficient) AND variation (e.g. standard deviation) or associated estimates of uncertainty (e.g. confidence intervals)
☐	☒	For null hypothesis testing, the test statistic (e.g. F, t, r) with confidence intervals, effect sizes, degrees of freedom and P value noted<br>*Give P values as exact values whenever suitable.*
☒	☐	For Bayesian analysis, information on the choice of priors and Markov chain Monte Carlo settings
☒	☐	For hierarchical and complex designs, identification of the appropriate level for tests and full reporting of outcomes
☐	☒	Estimates of effect sizes (e.g. Cohen's *d*, Pearson's *r*), indicating how they were calculated

Our web collection on statistics for biologists contains articles on many of the points above.

Software and code

Policy information about availability of computer code

Data collection	Data were reconstructed in CATMAID version 2021-12-21. Additional custom code is available on the Bagnall lab Github site: https://github.com/bagnall-lab/EM_analysis
Data analysis	Data were analyzed in Igor Pro 6, Matlab R2017a, and ZebraZoom 1.28.12.

For manuscripts utilizing custom algorithms or software that are central to the research but not yet described in published literature, software must be made available to editors and reviewers. We strongly encourage code deposition in a community repository (e.g. GitHub). See the Nature Portfolio guidelines for submitting code & software for further information.

Data

Policy information about availability of data

All manuscripts must include a data availability statement. This statement should provide the following information, where applicable:

- Accession codes, unique identifiers, or web links for publicly available datasets
- A description of any restrictions on data availability
- For clinical datasets or third party data, please ensure that the statement adheres to our policy

Quantification of hair cell to afferent and afferent to central target connectivity are provided (Supp. Data 1, 2). The electron micrograph image data generated in this study is hosted and publicly available at http://zebrafish.link/hildebrand16/data/vestibular_right

Field-specific reporting

Please select the one below that is the best fit for your research. If you are not sure, read the appropriate sections before making your selection.

☒ Life sciences ☐ Behavioural & social sciences ☐ Ecological, evolutionary & environmental sciences

For a reference copy of the document with all sections, see nature.com/documents/nr-reporting-summary-flat.pdf

Life sciences study design

All studies must disclose on these points even when the disclosure is negative.

Sample size	Sample size was n = 1 larval zebrafish for the EM dataset. This is the norm for large-scale serial-section electron microscopy reconstructions at this time. Within this fish we analyzed all utricular hair cells and afferents and central targets as described. For behavioral data, sample size was not predetermined but similar to those reported elsewhere in the literature. For behavioral data, sample sizes were not predetermined but involved two clutches (birth cohorts) for each experiment; data were only analyzed at the completion of all data collection. Sample sizes are similar to those reported in Tabor....Burgess J Neurophys 2014, which also examined Mauthner cell mediated escapes.
Data exclusions	No data were excluded unless adequate reconstruction could not be made (EM) or fish swam out of the field of view (behavior).
Replication	No replication of EM data due to only one animal. For behavioral data, each result included animals from two clutches (birth cohorts).
Randomization	Not relevant, behavioral data were acquired from two genetically distinct groups. N/a for the EM data with one animal.
Blinding	No blinding was possible due to obvious phenotype. However all behavioral data were analyzed with software, not scored by hand.

Reporting for specific materials, systems and methods

We require information from authors about some types of materials, experimental systems and methods used in many studies. Here, indicate whether each material, system or method listed is relevant to your study. If you are not sure if a list item applies to your research, read the appropriate section before selecting a response.

Materials & experimental systems

n/a	Involved in the study	
☒	☐	Antibodies
☒	☐	Eukaryotic cell lines
☒	☐	Palaeontology and archaeology
☐	☒	Animals and other organisms
☒	☐	Human research participants
☒	☐	Clinical data
☒	☐	Dual use research of concern

Methods

n/a	Involved in the study	
☒	☐	ChIP-seq
☒	☐	Flow cytometry
☒	☐	MRI-based neuroimaging

Animals and other organisms

Policy information about studies involving animals; ARRIVE guidelines recommended for reporting animal research

Laboratory animals	Danio rerio, 5-6 days post fertilization, wild-type animals and rock solo (otogelin mutant) as described in text.
Wild animals	Did not involve wild animals
Field-collected samples	Did not involve field samples
Ethics oversight	All experiments on animals were approved by the Washington University Institutional Animal Care and Use Committee.

Note that full information on the approval of the study protocol must also be provided in the manuscript.
