# Supplementary material for: Organization of the gravity-sensing system in zebrafish
Source: Nat Commun. 2022 Aug 27;13:5060. doi: 10.1038/s41467-022-32824-w (PMC9420129; doi:10.1038/s41467-022-32824-w)
Supplement: Supplementary file 11 — Source Data [file 41467_2022_32824_MOESM11_ESM.zip › JSON files/JSON file instructions.docx]

**Figures 1e, f, g; 2d, e; 3a; 4a; 5e; 6a**

Instructions for loading .json files

In browser, navigate to

<https://zf.hms.harvard.edu/hildebrand16/data/vestibular_right>

Click the “3D” button in top navigation panel


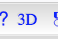


Click “Open JSON”, navigate to the .json file, and select it.


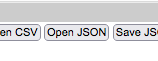


The file will load in the 3D viewer window. Zoom by scrolling with the mouse wheel, click and drag in the 3D view to rotate the view, and toggle various elements on/off in the neuron list below (e.g., toggle off the “meta” button to remove the red circles.)
