# Supplementary material for: Organization of the gravity-sensing system in zebrafish
Source: Nat Commun. 2022 Aug 27;13:5060. doi: 10.1038/s41467-022-32824-w (PMC9420129; doi:10.1038/s41467-022-32824-w)
Supplement: Supplementary file 11 — Source Data [file 41467_2022_32824_MOESM11_ESM.zip › Reconstruction files/Reconstruction files.pdf]

Source Data 3

This folder consists of neuronal reconstructions in the form of .swc files.
